# EspB and HtpG interact with the type III-A CRISPR/Cas system of *Mycobacterium tuberculosis*

**DOI:** 10.3389/fmolb.2023.1261613

**Published:** 2023-11-27

**Authors:** Mingmin Shi, Hongtai Zhang, Joy Fleming, Wenjing Wei, Hong Chen, Xiaowei Dai, Yi Liu, Chuanyou Li, Fanlei Ran, Zhilong Wu, Yaguo Wang, Xilin Zhang, Huizhi Zhang, Lijun Bi

**Affiliations:** ^1^ Foshan Fourth People’s Hospital, Foshan, China; ^2^ Key Laboratory of RNA Biology, State Key Laboratory of Biomacromolecules, CAS Center of Excellence in Biomacromolecules, Institute of Biophysics, Chinese Academy of Sciences, University of Chinese Academy of Sciences, Beijing, China; ^3^ Beijing Center for Diseases Prevention and Control, Beijing, China; ^4^ Biobank of Beijing Chest Hospital, Beijing Chest Hospital, Beijing Tuberculosis and Thoracic Tumor Research Institute, Capital Medical University, Beijing, China; ^5^ TB Healthcare Co., Ltd., Foshan, China; ^6^ Key Laboratory of Biomedicine, Innovation and Development Research Institute of International Eurasian Academy of Sciences, Beijing, China; ^7^ Guangzhou National Laboratory, Guangzhou, China

**Keywords:** CRISPR/Cas system, *Mycobacterium tuberculosis*, EspB, HtpG, Csm

## Abstract

**Introduction:** *Mycobacterium tuberculosis* (MTB) has a type III-A clustered regularly interspaced short palindromic repeat/CRISPR-associated protein (CRISPR/Cas) system consisting of a Csm1-5 and CRISPR RNA (crRNA) complex involved in the defense against invading nucleic acids. However, CRISPR/Cas system in the MTB still is clearly unknown and needs to be further explored.

**Methods:** In our work, two non-Cas system proteins EspB and HtpG protein were found and identified by LC-MS/MS. The effect of EspB and HtpG on Type III-A CRISPR/Cas System of *M. tuberculosis* was examined by using Plasmid interference assay and Co-immunoprecipitation analyses. We explored that EspB could interact with the crRNA RNP complex, but HtpG could inhibit the accumulation of the MTB Csm proteins and defense the mechanism of CRISPR/Cas system.

**Results:** The proteins ESAT-6 secretion system-1(Esx-1) secreted protein B (EspB) and high-temperature protein G (HtpG), which were not previously associated with CRISPR/Cas systems, are involved in mycobacterial CRISPR/Cas systems with distinct functions.

**Conclusion:** EspB is a novel crRNA-binding protein that interacts directly with the MTB crRNP complex. Meanwhile, HtpG influences the accumulation of MTB Csm proteins and EspB and interferes with the defense mechanism of the crRNP complex against foreign DNA *in vivo*. Thereby, our study not only leads to developing more precise clinical diagnostic tool to quickly detect for MTB infection, but also knows these proteins merits for TB biomarkers/vaccine candidates.

## 1 Introduction

Before the coronavirus disease 2019 pandemic, tuberculosis, caused by the ancient pathogen *Mycobacterium tuberculosis* (MTB), was the leading cause of infectious disease-related deaths, with approximately 10 million new cases and 1.4 million deaths worldwide in 2019 (Pai et al., 2016; Gordon and Parish, 2018; Khurana and Aggarwal, 2020).

Similar to other prokaryotes, MTB has a clustered regularly interspaced short palindromic repeat/CRISPR-associated protein (CRISPR/Cas) system involved in shielding cells against invasive DNA. The type III-A CRISPR/Cas effector complex is composed of proteins Csm1-5 and CRISPR RNA (crRNA), and the system has features atypical of other type III-A systems: mature crRNA in its crRNA-CRISPR/Cas protein complex is of the same length (∼71 nt) and appears to not be subject to 3′ end processing after Cas6 cleavage of repeat RNA (He et al., 2012; Makarova and Koonin, 2015; Tamulaitis et al., 2017; Wei et al., 2019). The crRNAs generated resemble mature crRNAs in type I systems, having both 5′ (8 nt) and 3′ (28 nt) repeat tags (Wei et al., 2019).

The pathogenic mechanisms of MTB are markedly different from those of other bacteria. Instead of exerting virulence via toxin production, the virulence factors of MTB play an important role in its pathogenicity (Forrellad et al., 2013; Bottai et al., 2014; Kroesen et al., 2019). It is the interplay between pathogen virulence factors and host tissue cell components that determines whether a disease will develop (Alderwick et al., 2015; Abrahams and Besra, 2021).

The CRISPR system of MTB includes two tandem CRISPR units, followed by nine consecutive *Cas* genes [Cas2, Cas1, Csm6, Csm5, Csm4, Csm3, Csm2, and Csm1 (Cas10) and Cas6]. The CRISPR/Cas system of MTB is classified as type III-A due to the presence of the signature protein Cas10 (He et al., 2012; Makarova and Koonin, 2015). Reports have suggested that CRISPR/Cas system proteins are involved in virulence (Lam et al., 2011; Li R. et al., 2016; Wong, 2017). Csm1, Csm3, Csm5, Csm6, and Cas6 are secreted and induce host immune responses (Jiao et al., 2021). In particular, Cas6, provokes interferon gamma (IFN-γ) release from the peripheral blood mononuclear cells of patients with active tuberculosis, and its deletion markedly attenuated virulence in a murine MTB challenge model. Recombinant MTBCas6 induces apoptosis of macrophages and lung fibroblasts, and interacts with the surface of cells in a caspase- and TLR-2-independent manner (Amaral et al., 2016; Jiao et al., 2021). Transcriptomic and signaling pathway studies using MTBCas6-stimulated THP-1 macrophages showed that MTBCas6 can upregulate the expression of genes associated with the nuclear factor kappa B pathway, leading to higher levels of interleukin (IL)-6, IL-1β, and tumor necrosis factor alpha release, which are cytokines that activate immune system cells in response to MTB infection (Khandelwal et al., 2011; Tanaka et al., 2016; Jiao et al., 2021).

CRISPR-Cas proteins can be influenced by or indirectly interact with non-Cas proteins (Yosef et al., 2011; Nuñez et al., 2016). In *Escherichia coli* (*E. coli*), high-temperature protein G (HtpG) deficiency can be suppressed by the expression of Cas3. The *E. coli* CRISPR system loses its suicidal activity against λ prophage and its ability to provide immunity from lysogenization in the absence of HtpG (Yosef et al., 2015; Nuñez et al., 2016). The heat-stable nucleoid-structuring protein, a global transcriptional repressor, can inhibit the CRISPR array and transcription of the cascade genes (*casABCDE*) to some extent as a negative regulator (Pul et al., 2010; Westra et al., 2010; Medina-Aparicio et al., 2011). LeuO, a LysR-type transcription factor, has been shown to derepress the transcription of *casABCDE12*, resulting in reduced CRISPR/Cas system activity (Westra et al., 2010; Medina-Aparicio et al., 2011). *Thermus thermophilus* Csm (TthCsm) can bind a nascent transcript in a transcription elongation complex that promotes tethering; although, there is no direct contact between TthCsm and RNA polymerase (RNAP) (Liu et al., 2019).

To date, there have been few reports on the involvement of non-Cas proteins in the MTB CRISPR/Cas system. Here, we present an investigation of the MTB CRISPR/Cas crRNA-Csm protein complex purified from cellular extracts using anion exchange, size-exclusion, and native-gel chromatography. ESAT-6 secretion system-1 (ESX-1) secretion system secreted protein B (EspB) in type III CRISPR/Cas systems has not been previously reported, and HtpG, a chaperone molecule that is required for CRISPR interference in *E. coli* (Yosef et al., 2011; Huang and Bao, 2016; Wong, 2017). In this work, EspB and HtpG were detected using the data of the purified crRNA-Csm protein complex obtained via mass spectrometry (MS). Further studies found that EspB is not only a novel crRNA-binding protein but also interacts directly with the crRNA ribonucleoprotein (crRNP) complex, which contains crRNA and Csm proteins. HtpG influences the accumulation of MTB Csm proteins and directly influences the defense mechanism of the CRISPR/Cas system against foreign DNA, although it does not interact with the crRNP complex. These findings aim at further supplementing and perfecting CRISPR/Cas System and explore more sensitive, efficient target protein as TB biomarkers for clinical applications.

## 2 Materials and methods

### 2.1 Bacterial strains and culture conditions

Experiments were performed with the MTB reference strain H37Rv, *M. bovis bacillus* Calmette-Geurin (BCG) (Pasteur), and its isogenic mutants ΔespB and ΔhtpG. Δ*espB* and Δ*htpG* were constructed as described previously (Jain et al., 2014). MTB strains were grown in Middlebrook 7H9 medium supplemented with 0.5% glycerol, 10% oleic acid-albumin-dextrose-catalase (Becton Dickinson, Franklin Lakes, NJ, United States) and 0.05% Tween-80 or on Middlebrook 7H10 agar (BD Difco Laboratories, Sparks, MD, United States) supplemented with 10% oleic acid-albumin-dextrose-catalase.

### 2.2 Isolation of the MTB crRNP complex

The crRNP complex was isolated using a strategy similar to that described by Wei et al. (2019). MTB cells were resuspended and disrupted in Fastprep-24 (MP Biomedicals, Santa Ana, CA, United States). The cell extract was subjected to ultracentrifugation, after which the supernatant was filtered twice with a 0.22 μm pore Millex filter unit (Millipore Sigma, Burlington, MA, United States) and loaded onto a 5 mL Q-Sepharose Fast Flow (GE Healthcare, Chicago, IL, United States) prepacked column. The proteins were eluted using a 0–1 M NaCl gradient. RNA was isolated from 200 μL of each fraction with TRIzol (Invitrogen, Carlsbad, CA, United States) according to the manufacturer’s instructions, and the fractions were analyzed via northern blotting and separated on 15% Tris-borate-ethylenediaminetetraacetic acid-urea gels. Blots were analyzed for the presence of spacer 1.04 to guide the purification process (Wei et al., 2019). The fractions, which had strong and clear signal for probes, were pooled, diluted in 50 mM Tris (pH 7.0) and 400 mM NaCl, and loaded onto a Superdex 200 10/300 GL column (GE Healthcare). Fractions from this column were selected based on signals obtained by northern blotting using a spacer 1.04 probe, pooled, and used in subsequent experiments. Native gel northern analysis using a spacer 1.04 probe was performed as described previously (Hale et al., 2009; Wei et al., 2019) to assess the mobility of crRNAs in the purified protein fraction. The protein bands containing the crRNP complex were then excised from the native gel, followed by in-gel trypsin digestion and identification by tandem MS.

### 2.3 Construction of recombinant proteins

The pMtbCsm1-6 plasmid, in which MTB csm2 was replaced with a homologous *M. Canettii* csm2 with an N-terminal His6 tag, was engineered to express the Csm complex. MTB csm1, csm3, csm4, csm5, and csm6 were codon optimized for expression in *E. coli*. The codon optimization sequence was previously described by Gruschow et al. (2019). Each csm gene had an independent T7 promoter, high-expression ribosome-binding site, and a T7 terminator, a strategy similar to that described by Ichikawa et al. (2017). The full csm fragment, containing csm1-csm6 together with their respective T7 promoter and T7 terminator, was synthesized by Sangon Biotech (Shanghai, China) and cloned into a pET-28a vector by Seamless Cloning (GeneArt^®^ Seamless Cloning and Assembly; Thermo Fisher Scientific, Waltham, MA, United States). A pMtbCas6-crRNA plasmid was constructed to express Cas6 and crRNA. The pCDFDuetTM vector, compatible with the pET-28a vector, was used to co-express Cas6. A CRISPR sequence (3R2S) containing three repeats and two spacer 1.01 units from the CRISPR 1 locus (Supplementary Table S1) was synthesized by Sangon Biotech and cloned into a pCDFDuetTM vector together with cas6, and espB and htpG were amplified from the MTB H37Rv genome and cloned into pET22b and pACYADuet-1 vectors separately.

### 2.4 Protein expression and purification

Condon-optimized MtbMBP-csm1 (Rv2823c), Mtbcsm2 (Rv2822c) Mtbcsm3 (Rv2821c), Mtbcsm4 (Rv2820c), Mtbcsm5 (Rv2819c), and Mtbcsm6 (Rv2818c) were cloned into pET-28a vector, separately, to generate individual Csm-expressing plasmids. Individual Csm plasmid (pCsm1, pCsm3, pCsm4, pCsm5, pCsm6) and HtpG was each transformed into *E. coli* BL21 (DE3) (Novagen) grown at 37°C with 50 μg/ml of kanamycin selection. Protein expression was induced by adding 0.3 mM IPTG (isopropyl β-D-1-galactopyranoside) at OD600 = 0.6 at 16°C overnight. Cells were collected and resuspended in lysis buffer [20 mM Tris-HCl, pH 7.5, 300 mM NaCl, 20 mM imidazole, 5% (v/v) glycerol], and then lysed by sonication. Supernatant by centrifugation was loaded onto HisPur Ni-NTA resin (Thermo Scientific) and the bound protein was washed by washing buffer [20 mM Tris (pH7.5), 300 mM NaCL, 100 mM imidazole, 5% (v/v) glycerol] and eluted by elution buffer containg 20 mM Tris (pH7.5), 300 mM NaCL, 500 mM imidazole, 5% (v/v) glycerol. Individual Csm proteins (MtbCsm1, MtbCsm3, MtbCsm4, MtbCsm5, MtbCsm6) and HtpG was separately further purified by size-exclusion chromatography (Superdex 75 10/300 GL, GE Healthcare) in a buffer containing 20 mM Tris-HCl (pH 7.5), 300 mM NaCl, 5% (v/v) glycerol. Fractions containing the Csm complex and HtpG were identified by Coomassie Brilliant Blue G250 staining SDS-PAGE, flash-frozen with nitrogen and collected at −80°C. MtbCsm2 was purified from inclusion bodies of *E. coli*. The purification process was carried out following established procedure (Wang et al., 2009; Zhang et al., 2023), including protein inclusion body washing, inclusion body dissolving, urea gradient dialysis and protein refolding. The protein supernatant was pooled for further studies.

The pMtbCsm1-6, pMtbCas6-crRNA, pEspB, and pHtpG plasmids were cotransformed into *E. coli* BL21 (DE3) cells (Novagen). When the optical density at 600 nm reached 0.6, protein expression was induced with 0.1 mM isopropyl-1-thio-β-D-galactopyranoside for 5 h at 28°C. Cells producing the His6-tagged Csm complex were resuspended in 25 mM Tris-HCl (pH 8.0) containing 300 mM NaCl and lysed using a high-pressure cell disrupter. After centrifugation, the supernatant was applied to a Ni Sepharose column (GE Healthcare) pre-equilibrated with 25 mM Tris-HCl (pH 8.0) containing 0.3 M NaCl and 20 mM imidazole. The bound proteins were eluted with buffer containing different concentrations of imidazole (20, 50, 100, and 500 mM). Target fractions were then collected and analyzed using sodium dodecyl sulfate-polyacrylamide gel electrophoresis (SDS-PAGE) and Coomassie Brilliant Blue G250 staining.

### 2.5 Protein-protein/RNA interaction

A biolayer interferometry experiment was carried out at 25°C on Octet RED96e (GE Healthcare) to test the interaction of protein-RNA interaction. To measure and compare the strength of the interactions of MTB EspB and HtpG with different RNAs, 3′-biotinylated RNAs were immobilized on a streptavidin-coated sensor chip, and protein (EspB/HtpG) was injected at creasing concentrations over the surface (500–16,000 nM). Surface plasmon resonance (SPR) experiments were carried out on Biacore 8K (GE Healthcare) at 25°C to test protein-protein interactions. The ligand was covalently immobilized on the dextran surface of a CM5 optical chip. The analyte samples were injected at different concentrations (25–3,200 nM) over the surface at a flow rate of 30 μL/min for 2 min. All experiments were repeated at least thrice.

### 2.6 Flow cytometry

Plasmids containing the C-termini of *csm*, *htpG*, and *espB* fused with enhanced green fluorescent protein (eGFP) were transformed into BCG and BCGΔhtpG, respectively for constitutive expression system. Samples for flow cytometry analysis were washed with 0.22 μm-filtered 1× phosphate-buffered saline (PBS). A 200 μL aliquot of each sample was transferred to a fluorescence-activated cell sorting analysis tube, and bacterial cells were injected into the flow cytometer within a few minutes after resuspension. A FACScalibur flow cytometer (Becton Dickinson) equipped with a 15-mW, air-cooled argon ion laser was used as the excitation light source (488 nm), and fluorescence (500–549 nm) was detected using a fluorescence detector. The analysis of each sample was stopped after 100,000 cells had been counted.

### 2.7 Plasmid interference assay

Competent *E. coli* BL21 strains containing a *csm* module (Mtb*csm*1-6, Mtb*cas6* and crRNA 1.01 spacer) were transformed with 100 ng of either the target or nontarget plasmid. Heat shock transformation was performed, and 800 μL of Luria-Bertani medium was immediately added to the competent cells (50 μL), and incubated with shaking at 200 rpm at 37°C for 45 min. Three sequential 1:10 dilutions was prepared for each transformation, and 20 μL of each dilution was spread onto Luria-Bertani agar plates. Plates were incubated overnight at 37°C, and images of the colonies were captured on a Gel Doc XR (Bio-Rad, Hercules, California, United States) using white light. Assays were performed in triplicate and analyzed using two-way analysis of variance.

### 2.8 Co-immunoprecipitation

The cells were harvested and lysed in PBS. Lysates were incubated with the corresponding antibody or nonspecific immunoglobulin G antibody (negative control) at 4°C. Protein G agarose beads were then added to the mixtures and incubated for 2 h on a rotatory platform at 4°C. Bound proteins were eluted by adding SDS-PAGE treatment buffer.

### 2.9 Western and Northern blotting

Samples were separated on 12% SDS-PAGE and then transferred to nitrocellulose membranes. The membranes were blocked with 1x PBS with Tween 20 (PBST) with 5% (w/v) nonfat milk for 1 h at room temperature and then incubated overnight at 4°C with a 1:1000 dilution of the appropriate antiserum. After washing the membranes with PBST three times, bound primary antibodies were detected using an Amersham Imager 600 detection system (GE Healthcare).

DNA oligonucleotide (Fourth spacer from the MTB CRISPR1 locus) probes were end-labeled in a 20 μL kinase reaction containing 10 pmol γ-32P-adenosine triphosphate and 20 U T4 polynucleotide kinase (New England Biolabs, Ipswich, MA, United States) at 37°C for 1 h. Ten micrograms of total RNA were separated on 10% or 15% denaturing acrylamide gels and electroblotted onto Amersham Hybond-XL membranes (GE Healthcare, Waukesha, WI, United States) (Wei et al., 2019). After Pre UV-Cross-linking, the membranes were incubated with labeled probes for 3–6 h in Amersham Rapid-hyb Buffer (GE Healthcare), washed, and exposed to Kodak film over night or for 3 days. All experiments were repeated at least three times (Wei et al., 2019).

### 2.10 Liquid chromatography (LC)-MS/MS analysis and data processing

Lanes of interest were manually cut out of the preparative gel. After de-staining, dithiothreitol reduction and alkylation of iodoacetamide, trypsin digestion were performed as described elsewhere (Wilm et al., 1996; Fandino et al., 2005). Peptide solutions were extracted three times with a solution containing 60% acetonitrile (ACN) and 0.1% formic acid. Peptides were analyzed by nanoLC-LTQ-MS/MS (ThermoFinnigan, San Jose, CA) equipped with a nano-electrospray ion source, separated with an online reversed-phase LC column (100 μm × 150 mm, 3 μm, Pepmap C18) and gradually eluted with a 0%–100% gradient of buffer B (ACN: water: formic acid, 80:19.9:0.1) in buffer A (ACN: water: formic acid, 2:97.9:0.1) for 70 min at flow rate of 500 nL/min. MS parameters included 2.1 kV electrospray voltage, 300–1,800 m/z range, 90 s exclusion duration and 210°C source temperature.

To identify accurate peptides, LC-MS/MS data was evaluated with Bioworks software (version 3.3.1, Thermo Fisher Sceintific) software and sequences identical to the peptides used to generate the LC-MS/MS data were searched in the National Center for Biotechnology Information database. The search parameters were as follows: species: MTB (H37Rv); digest: trypsin; sites: two missed cleavage sites per peptide allowed; mass error: precursor ion <3 Da and fragment ion <1 Da. The search was set with cysteine carbami-domethylation as the fixed modification and methionine oxidation as the variable modification. The filtering criteria of the MS/MS search result were as follows: 1) The correlation coefficients (Xcorr) of peptides with +1, +2, and +3 charges after complete digestion were greater than 1.90, 2.5, and 3.75, respectively; 2) Sp value of ≥500, Rsp value of ≤5; 3) Each identified protein containing at least two specific peptide segments to support. Results were manually verified and validated.

## 3 Results

### 3.1 Identification of EspB and HtpG in an isolated MTB crRNP complex

**FIGURE 1:**
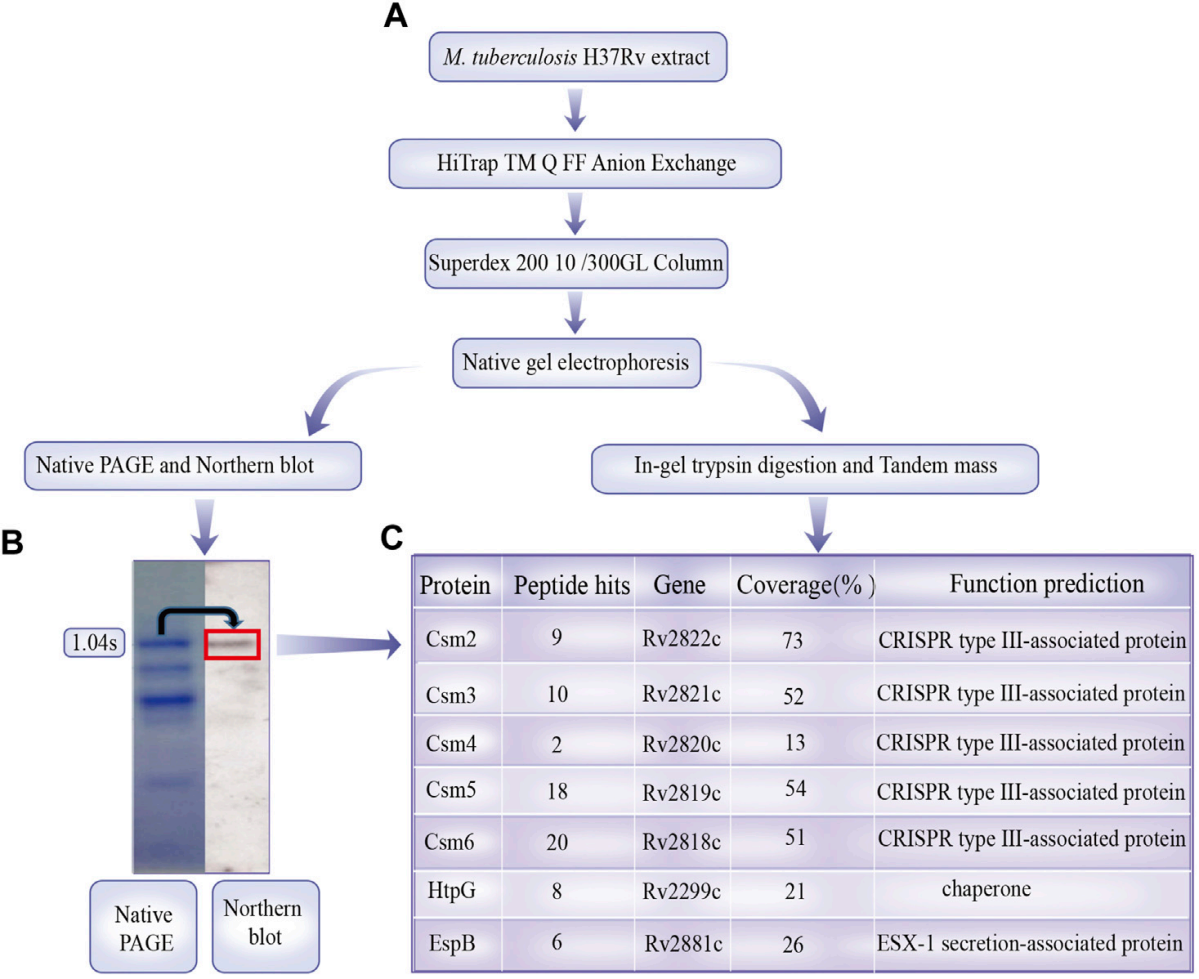
*Mycobacterium tuberculosis* crRNA ribonucleoprotein (crRNP) complex purification and identification. **(A)** *M. tuberculosis* H37Rv cell extract was separated by Q-Sepharose anion exchange chromatography, and crRNA in the fractions were examined via Coomassie Brilliant Blue G250 staining and northern analysis using probes against spacer 1.04. The band of the crRNP complex on the gels is indicated with dotted boxes. **(B)** Coomassie Brilliant Blue G250-stained bands were subjected to in-gel trypsin digestion and identified by tandem mass spectrometry. **(C)** Sequence coverage and unique peptides for proteins identified with 99% confidence are shown. Annotations of proteins are from the National Center for Biotechnology Information database.

A summary of results are displayed in Supplementary Table S1. CRISPR/Cas proteins may act together with non-Csm proteins in certain biological functions. For example, *T. thermophilus* (type III-A) crRNP interacts with RNA and DNA via RNAP to cleave RNA in an RNAP-bound transcription bubble (Liu et al., 2019). Csm proteins Csm1-5 form a complex with mature crRNAs that are involved in the cleavage of nucleic acids (Staals et al., 2014; Tamulaitis et al., 2017). To investigate whether other proteins interact with the MTB crRNP complex (Csm1-5 and crRNA) *in vivo*, we isolated the crRNP complex using the strategy employed by Wei et al. (2019). Northern blotting with an anti-1.04 probe (fourth spacer from the CRISPR1 locus) was used to locate the purified mature crRNP complex on the native gels (Figures 1A, B). The band corresponding to the crRNP complex was gel digested, and the tryptic peptides of the complexes was identified by the nano LC-TLQ-MS/MS. The significant peptide including duplicate sequences from separate MS/MS spectra was detected and shown the sequences of the protein coverage regions. Tryptic digestion provided a high number of cleavages and produced long peptides resulting in a high percentage (32%∼75%) of sequence coverage. The MS/MS search included seven proteins (Csm 2-6, Chaperone htpG, and ESX-1 secretion-associated protein EspB) were involved in the MTB H37Rv strain; however, Csm1 was lost (Figure 1C; Supplementary Table S1). Although Csm4 protein was identified from only two peptides, the data was reliable since the peptide spectra was manually validated; therefore, protein identification was effective and sufficient.

Csm6 did not interact with the crRNP complex; however, its RNase activity was stimulated by short cyclic oligoadenylate signaling molecules generated by the crRNP complex, Csm1, upon target RNA recognition. Csm1 was not identified, possibly owing to partial degradation during isolation.

### 3.2 EspB can bind MTB crRNA

**FIGURE 2:**
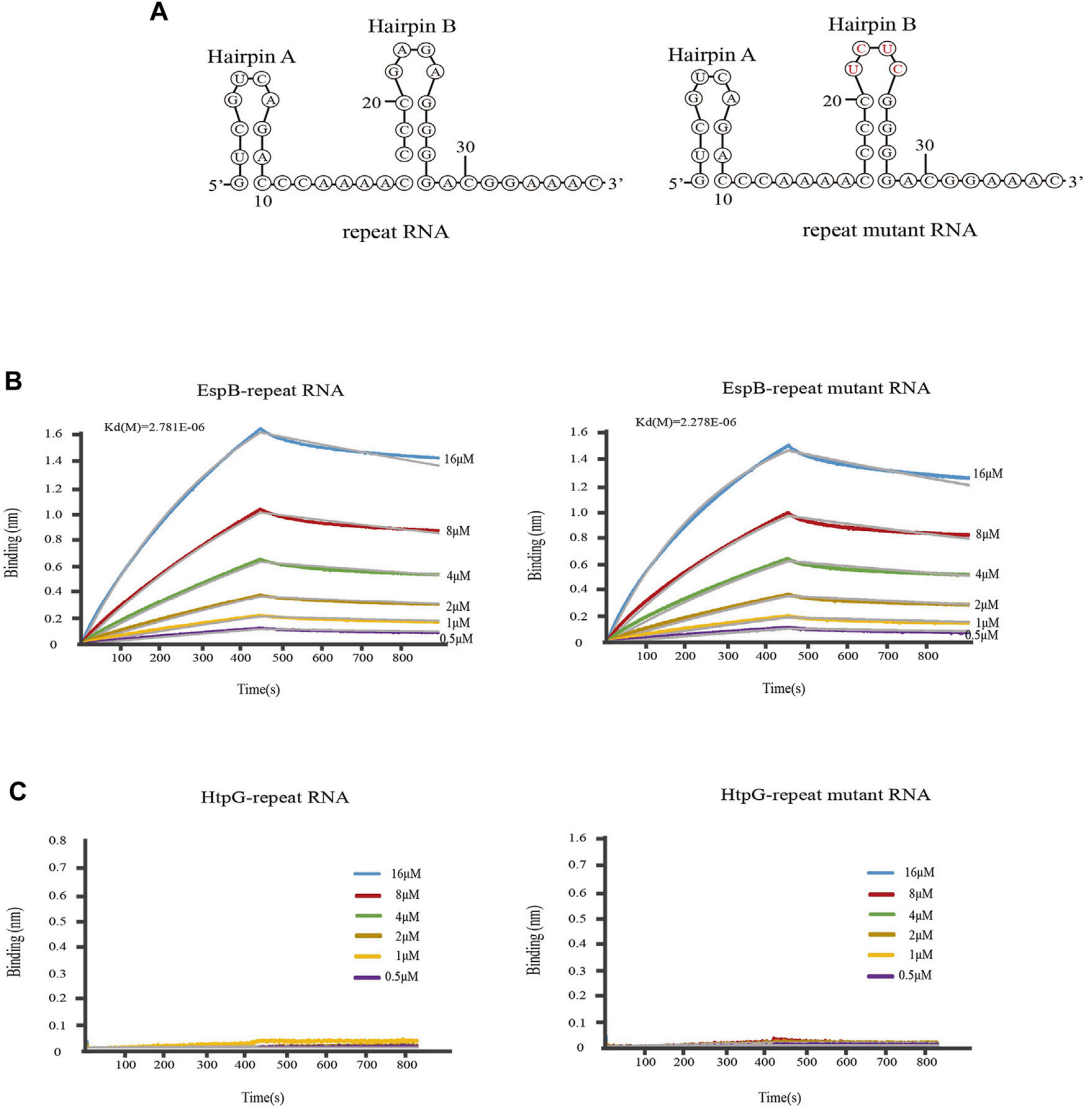
Kinetic analysis of the binding of EspB and HtpG to *M. tuberculosis* crRNA. **(A)** The secondary structure of Repeat RNA and mutant RNA. **(B)** Kinetic analysis of the binding of EspB to crRNA. **(C)** Kinetic analysis of the binding of HtpG to crRNA. Biolayer interferometry experiment was carried out at 25°C on an Octet Red96 (Fortebio) instrument to detect the binding of EspB and HtpG proteins to repeat RNA or mutant repeat RNA, in which bases in the 5′ end loop were mutated (GAGA to UCUC). The colored lines represent the specific binding profiles of various concentrations of EspB to 3′-immobilized biotinylated RNAs.

To investigate whether EspB and HtpG could bind crRNA and participate in the crRNP complex, we first explored whether HtpG and EspB interacted with crRNA using BLI. In brief, the results indicated that EspB could bind to MTB crRNA 1.01 (Figure 2B). Relevant kinetic paramenters calculated by BLI was listed in Supplementary Table S3.

Mature crRNAs in MTB are of the same length (∼71 nt), and their structure (intact spacer flanked by 3′ and 5′ repeat sequence tags, with the 3′ tag forming a hairpin structure) closely resembles that of type I system mature crRNAs. The sequence or structure of the 3′ hairpin B in the repeat RNA of MTB crRNAs is important for MTBCas6 recognition or cleavage of the substrate RNA. The accuracy of mature crRNA cleavage in MTB depends on the presence of the 3′ hairpin B structure and its base sequence (Zhang et al., 2021). We investigated whether the binding ability of EspB to crRNA 1.01 was influenced by the 3′ hairpin B structure and its base sequence by mutating GAGA to UCUC within the 3′ hairpin B [Seen the secondary structure in Figure 2A (Zuker, 2003)]. The results showed that EspB could bind repeat RNA sequence and binding ability was not affected by mutated sequences (Figure 2B). However, Figure 2C showed that HtpG could not directly interact with repeat RNA or repeat mutant RNA. Consistent with this result, Yosef et al. (2011) only found that HtpG was essential for the function level of CRISPR system.

### 3.3 EspB interacts with the MTB crRNP complex

**FIGURE 3:**
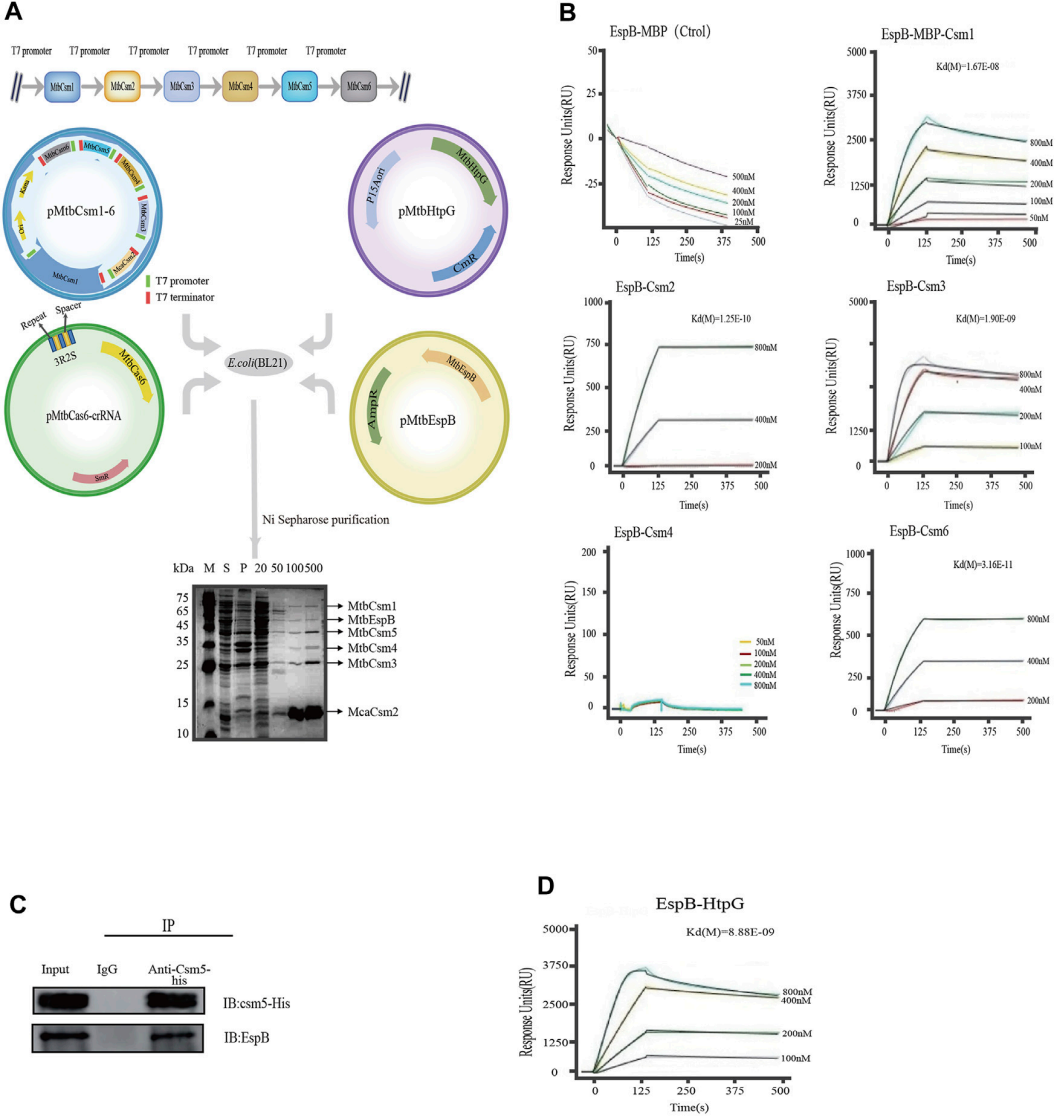
Kinetic analysis of the binding of EspB to Csm proteins. **(A)** Interaction between HtpG, EspB, and the crRNP complex. pMtbCsm1-6: A T7 promoter (green) and T7 terminator (red) were engineered upstream and downstream of each Csm element, respectively; pMtbCas6-crRNA: The blue and yellow rectangles indicate the repeats and spacers elements, respectively, from the native CRISPR locus 1 of the strain H37Rv; pEspB: espB gene was amplified and cloned into pET-22b(+); pHtpG: htpG gene was amplified and cloned into pACYCDuet-1. The four plasmids (pMtbCsm1-6, pMtbCas6-crRNA, pHtpG, and pEspB) were cotransformed into *E. coli* BL21(DE3) cells (Novagen) for purification. Lane M: Color Prestained Protein Marker; Lane S: Supernatant of cell lysates; Lane P: Precipitate of cell lysates; Lanes 20, 50, 100, and 500: Fractions eluted with elution buffer containing 20-, 50-, 100-, and 500-mM imidazole. **(B)** Kinetic analysis of the binding of EspB to Csm(s) via surface plasmon resonance *in vitro*. The colored lines represent the specific binding profiles of various concentrations of Csm to EspB protein surface. Data were globally fitted to a 1:1 mass transport model using BIAevaluation 3.0 software. **(C)** Co-immunoprecipitation assay for verification of the interaction between EspB and Csm5 *in vivo*. **(D)** Kinetic analysis of the binding of EspB to HtpG via surface plasmon resonance *in vitro*. The colored lines represent the specific binding profile of various concentrations of HtpG to EspB protein surface. Data were globally fitted to a 1:1 mass transport model using BIAevaluation 3.0 software.

We first investigated whether HtpG and/or EspB interacted with the crRNP complex *in vivo*. We simultaneously expressed various proteins in *E. coli* BL21 (DE3) competent cells using the following plasmids: 1) codon-optimized MTB *csm* genes: *csm1*-*His*-tagged *mca2*-*csm3*-*csm4*-*csm5*-*csm6* genes [MTBCsm2 was replaced by its homologue from *M. canettii* (Supply and Brosch, 2017) (McaCsm2; 71% amino acid identity), as the expression and purification of MTBCsm2 was problematic]; 2) *cas6* repeat-spacer sequence; 3) *espB gene*; and 4) *htpG gene* (Figure 3A). Cell lysates were purified with HisPur Ni-NTA resin targeting the 6-His tag on Mca2. Proteins were eluted at different concentrations of imidazole (50, 100, and 500 mM), and six bands (corresponding to different elution concentrations) were observed on SDS-PAGE. Nano LC-TLQ-MS/MS also identified two non-Cas system proteins, namely, EspB and HtpG, indicating that EspB interacted with the MTB crRNP complex *in vitro* (Supplementary Table S1).

Subsequent SPR results confirmed that EspB could interact with Csm1, Csm2, Csm3, and Csm6; however, we were unable to detect any interaction between EspB and Csm4 *in vitro* (Figure 3B). As Csm5 is unstable when released from the complex *in vitro*, we performed a co-immunoprecipitation assay in *M. bovis* BCG, whose CRISPR/Cas system is similar to that of MTB to validate its interaction with EspB. Western blotting demonstrated that EspB interacted with Csm5 *in vivo* (Figure 3C).

Even though HtpG could not interact with the crRNP complex, SPR experiments showed that HtpG interacted with EspB and Csm3 *in vitro* (Figure 3D; Supplementary Figure S1). The kinetic paramenter analysis was listed in Supplementary Table S4.

### 3.4 HtpG influences the accumulation of Csm proteins

**FIGURE 4:**
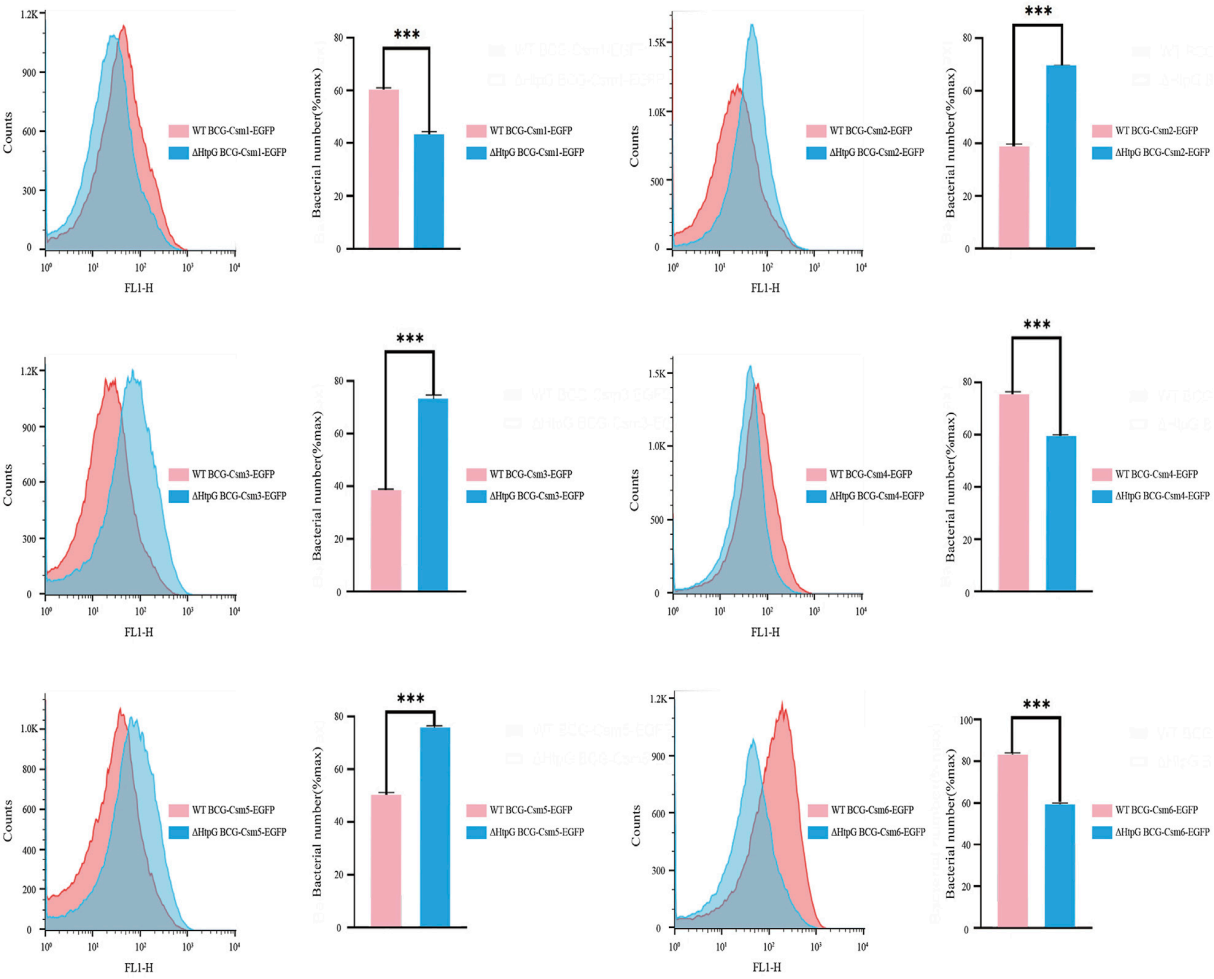
Effect of HtpG on the accumulation of *M. tuberculosis* Csm proteins. Counts: Number of enhanced green fluorescent protein-expressing cells. For each measurement, 100,000 events were collected. The measured fluorescence intensities represent the expression level of corresponding Csm proteins. ΔHtpG BCG: HtpG deficiency in *M. bovis bacillus* Calmette-Guérin.

As HtpG is essential for maintaining functional levels of Cas3 in *E. coli* (type I CRISPR/Cas system) (Yosef et al., 2011), we hypothesized that its expression might also affect the accumulation of Csm proteins in *Mycobacteria*. We transformed plasmids carrying *csm1*-e*gfp*), *csm2*-*egfp*, *csm3*-*egfp*, *csm4*-*egfp*, *csm5*-*egfp*, and *csm6*-*egfp* into wild-type *M. bovis* BCG and BCGΔ*htpG*, and eGFP intensities were measured using flow cytometry to determine the accumulation of Csm1-6 proteins. The results showed that Csm2 and Csm3 levels increased while Csm1, Csm4 and Csm6 decreased in both BCGΔ*htpG* relative to the wild-type control. These results indicate that *htpG* can influence the accumulation of Csm proteins (Figure 4).

However, when we transformed the *espB*-*egfp* plasmid into wild-type BCG and BCGΔ*htpG*, we obtained colonies of BCGΔ*htpG* overexpressing *espB*-*egfp* but not wild-type BCG overexpressing *espB*-*egfp.* These results indicate that overexpression of EspB can lead to the death of wild-type *M. bovis BCG*, while loss of *htpG* (BCGΔ*htpG*) confers a higher survival capability against EspB accumulation.

### 3.5 HtpG overexpression inhibits MTB CRISPR/Cas defense against foreign DNA

The MTB CRISPR/Cas system (type III-A), the crRNP complex, provides resistance against invasive phages and plasmids. Since EspB is involved in the MTB EspB-crRNP complex and HtpG overexpression affects the accumulation of Csm proteins, we investigated whether EspB and HtpG overexpression also influences the efficiency of MTB CRISPR/Cas anti-plasmid immunity. We simultaneously transformed foreign DNA from four plasmids [two expression plasmids containing *csm1*-*His*-tagged *mca2*-*csm3*-*csm4*-*csm5*-*csm6* and *cas6* + crRNA 1.01 (the first spacer in the MTB CRISPR1 locus), an artificial foreign plasmid designed to target crRNA 1.01, and a plasmid containing non-target crRNA 1.01 as a control] into *E. coli* BL21 (DE3) competent cells. The transformed plasmids containing non-target crRNA 1.01 were unaffected by the MTB Csm-crRNA complex; however, plasmids targeting crRNA 1.01 could not be transformed into *E. coli* BL21 (DE3) cells and no colonies were present on the culture plates. These results indicate that the reconstituted crRNA-Csm complex is active in defense and can provide immunity against certain foreign nucleic acids.

**FIGURE 5:**
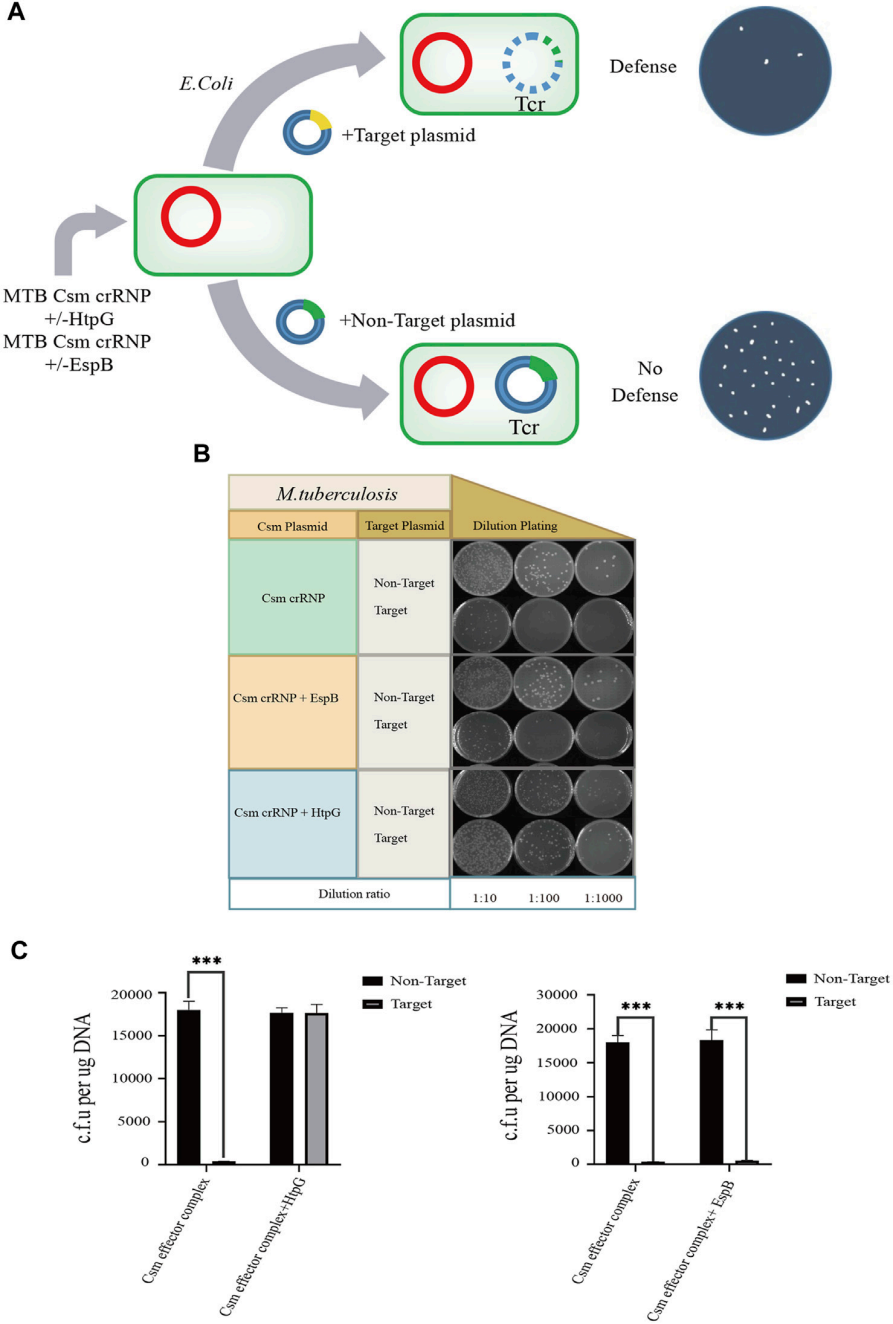
Effect of HtpG and EspB on *M. tuberculosis* type III-A immunity. Three different modules for expressing the MTB crRNP complex, MTB crRNP complex and EspB, or MTB crRNP complex and HtpG (red; kanamycin selectable) were transformed into *Escherichia coli* together with target (yellow; tetracyline) and nontarget plasmids (green; tetracycline). **(A)** The target plasmid contains a sequence (yellow) that is complementary to expressed crRNA, while the nontarget plasmid contains a sequence (green) that lacks crRNA homology. **(B)** Serial ten-fold dilutions of transformed cells were spotted onto plates containing kanamycin and tetracycline, and CRISPR/Cas-mediated plasmid loss (defense) is indicated by a reduction in colonies. **(C)** Effect of HtpG/EspB region on the plasmid transformation efficiency.

Next, we overexpressed EspB and HtpG in our recombinant *E. coli* BL21 (DE3) MTB CRISPR/Cas complex-containing cell line. When the artificial foreign plasmid targeting crRNA 1.01 was transformed into these cells (Figure 5A), EspB overexpression did not affect the efficiency of plasmid transformation. However, HtpG overexpression significantly inhibited the ability of the MTB crRNP complex to protect against foreign DNA (Figures 5B, C). To check the HtpG overexpressed in *E. coli* BL21(DE3) MTB CRISPR/Cas system, HtpG was identified and confirmed by LC-MS/MS (Supplementary Table S2). These findings done in this work may provide a useful guideline for clinical application and has important practical values.

## 4 Discussion and conclusion

**FIGURE 6:**
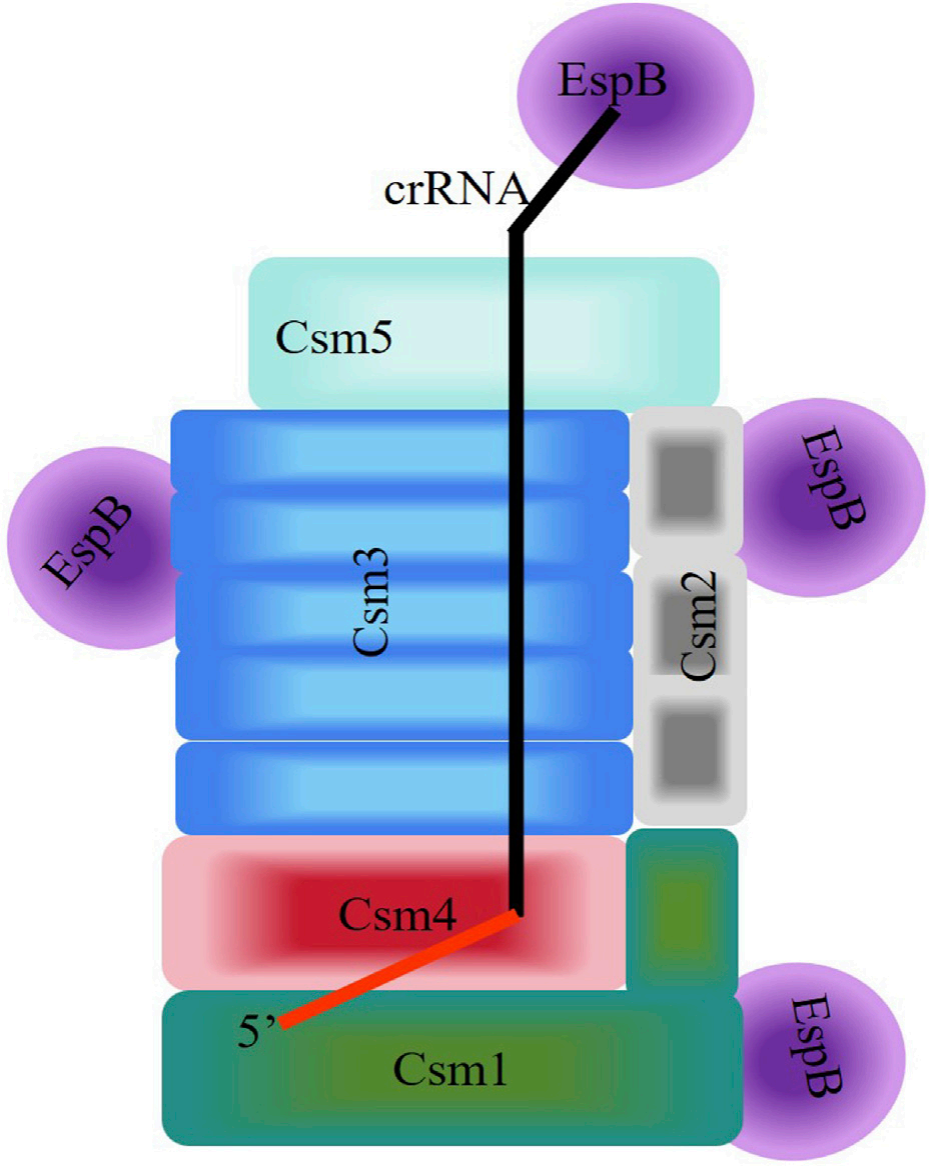
Schematic diagram of the interaction between EspB and the Csm complex of the Type III-A CRISPR/Cas System of Mycobacterium tuberculosis. Homologous subunits are depicted by the same color. The 5′-handle of crRNA in the Csm complexes is shown in red.

The canonical involvement of CRISPR/Cas proteins in type III CRISPR defense systems against invading nucleic acids has been extensively studied (Hale et al., 2009; Staals et al., 2014; Li Y. et al., 2016). However, few non-Cas proteins have been reported to directly interact with crRNPs (type III-A). Thus, we explored *non-Cas* genes associated with the MTB CRISPR system (Seen Figure 6). Additionally, the interactions of the proteins EspB and HtpG were identified by MS during the isolation of the MTB CRISPR/Cas complex, with the MTB crRNP complex. We showed that EspB and HtpG had distinct roles and interactions with the crRNP. EspB is a novel crRNA-binding protein that interacts directly with the crRNP complex. Meanwhile, HtpG cannot bind crRNA and interacts with the crRNP complex. The validity of HtpG overexpression was identified by SDS-PAGE analysis and LC-MS/MS (Supplementary Table S2; Supplementary Figure S2), but HtpG was not co-purified with crRNPs, maybe it was not expressed in this co-expression system or not present in the eluted fractions. Even so, HtpG overexpression could effectively inhibits the defense mechanism of CRISPR/Cas against foreign DNAs, possibly since MTB Csm protein accumulation is influenced by HtpG.

We isolated the crRNP complex as described by Hale et al. (2009), who identified seven Cas proteins in the *Pyrococcus furiosus* type III-B crRNP complex (Cmr1-1, Cmr1-2, Cmr2, Cmr3, Cmr4, Cmr5, and Cmr6). However, they did not report the presence of non-Cas proteins. MS of the MTB crRNP complex isolated here included four Csm proteins from the crRNP complex (Csm2, Csm3, Csm4, and Csm5, but not Csm1), and three additional proteins, namely, Csm6, EspB, and HtpG. It is likely that the isolated MTB crRNP complex not only contains proteins that directly interact with crRNP complexes to form a novel stable complex but also includes proteins that may be involved in CRIPSR/Cas defense. For example, the presence of Csm6 was identified by MS. Csm6, a Higher Eukaryotes and Prokaryotes Nucleotide-binding family ribonuclease, reportedly does not directly interact with the crRNP complex; however, its RNase activity is stimulated by short cyclic oligoadenylate signaling molecules generated by the crRNP complex Csm1 upon target RNA recognition, and its ribonuclease activity is required for antiplasmid immunity in type III-A CRISPR/Cas systems (Hale et al., 2009; Tamulaitis et al., 2017; Foster et al., 2019). Csm1 was not identified, possibly owing to degradation to the greatest extent.

EspB, an important modulator with strong links to the innate immunity and virulence of MTB (McLaughlin et al., 2007; Chen et al., 2013), is mainly present in pathogenic bacteria, such as *Mycobacterium* spp., enterohemorrhagic *E. coli*, *enteropathogenic E. coli*, and *Citrobacter rodentium* (Rabinovitz et al., 2016; Baumann et al., 2018). It is secreted via the MTB type VII secretion system (Simeone et al., 2009), and similar to other type VII secretion system proteins, such as ESAT-6, CFP-10, EspA, EspC, and EspR, it is an important secreted virulence factor (Renshaw et al., 2002; Guinn et al., 2004; Raghavan et al., 2008; Smith et al., 2008). We previously demonstrated that Csm1, Csm2, Csm3, Csm5, Csm6, and Cas6 are secreted antigens (Jiao et al., 2021) and Cas6 plays a particularly important role in virulence. Cas6 can induce macrophage apoptosis and activate the macrophage nuclear factor kappa B signaling pathway, leading to the release of cytokines important for immune cell activation. Furthermore, CRISPR/Cas proteins are involved in specific functions associated with pathogenic bacterial species. Our results showed that EspB directly interacted with the crRNP complex to form an EspB-crRNP complex; however, EspB did not influence the protective ability of the crRNP complex against foreign nucleic acids. It is likely that EspB has implications for the secretion of Csm proteins and noncanonical roles that the CRISPR/Cas system plays in virulence.

In the EspB-crRNP complex, EspB not only interacts directly with Csm proteins, but also binds crRNA. It is known that EspB plays an important role in host immune responses and is involved in the suppression of autophagosome formation, IFN-γR1 downregulation, and inhibition of IFN-γ-activated phosphorylation of signal transducer and activator of transcription 1 (Junqueira-Kipnis et al., 2006; Huang and Bao, 2016); however, the mechanism involved in MTB virulence remains largely unclear. Characterization of EspB as a secreted RNA-binding protein and the EspB-crRNP complex will provide novel insights into the role of EspB or the CRISPR/Cas system in MTB virulence.

HtpG was also detected by MS in the isolated crRNP complex; however, we were unable to identify the interaction of HtpG with the crRNP complex in pull-down experiments in *E. coli*, possibly due to a weaker interaction with some Csm proteins or the 6xHis tags on Csm2. We identified the interaction of HtpG proteins with EspB and Csm3 *in vitro* via SPR, which may be why HtpG was identified from the isolated crRNP complex in MTB.

In *E. coli*, when HtpG was deficient, higher levels of Cas3 protein, which is essential for immunity against foreign DNA, accumulated, and the defense mechanism of the CRISPR/Cas system against foreign nucleic acids was lost (Yosef et al., 2011). It is highly likely that the disproportionality of the accumulation of Cas proteins in the CRISPR/Cas system disturbs the efficiency of defense. In *Mycobacteria*, the deficiency of HtpG regulated the accumulation of Cas proteins to a greater extent than in *E. coli*, lowered the accumulation of Csm1, Csm4, Csm5, and Csm6, and increased the accumulation of Csm2 and Csm3. Along with HtpG overexpression inhibiting the MTB CRISPR/Cas defense against foreign DNA, these results indicate that HtpG as a modulator is essential for maintaining the function of the CRISPR/Cas system. We speculated that the lack of HtpG influences the appropriate accumulation of Csm proteins in the MTB crRNP complex, possibly resulting in the destruction of the assembling proportion of the MTB crRNP complex and dysregulation of the CRISPR system.

EspB and HtpG are involved in completely distinct functions associated with the CRISPR/Cas system. However, since overexpression of EspB leads to the death of wild-type *M. bovis BCG* while loss of *htpG* confers a higher survival capability survival capability, and HtpG/EspB interaction, this implies a relationship between HtpG and EspB associated with the CRISPR/Cas system.

Taken together, these biochemical assays suggests the speculative model of the interaction for assembly between EspB and the Csm complex in Type III-A CRISPR/Cas System of *M. tuberculosis* (Figure 6). But in order to clarify the more systemic mechanism, we still go on exploring on CRISPR/Cas System.

In conclusion, we reported the interaction of two non-Cas proteins, HtpG and EspB, with the MTB crRNP complex, and their distinct roles in the CRISPR/Cas system. HtpG did not interact with the MTB crRNP complex; however, it was involved in the canonical defense against foreign nucleic acids. Although EspB interacted directly with the MTB crRNP complex, it did not participate in the defense function of the CRISPR/Cas system. EspB may be involved in noncanonical roles, as we have previously reported that some of the CRISPR/Cas proteins with which they interact are secreted and involved in MTB virulence. Our results not only provide further insight into the role of non-Cas proteins in the defense function of the MTB CRISPR/Cas system, but may also elucidate the association of CRISPR/Cas proteins with virulence.

## Data Availability

The original contributions presented in the study are included in the article/Supplementary Material, further inquiries can be directed to the corresponding authors.

## Supplementary material

SUPPLEMENTARY TABLE S2 Tandem mass spectrometry of HtpG overexpression. HtpG overexpressed in recombinant E. coli BL21 (DE3) MTB CRISPR/Cas complex-containing cell line and identified by tandem mass spectrometry. The HtpG overexpression was subjected to in-gel trypsin digestion and identified by tandem mass spectrometry. Sequence of unique peptides for proteins identified with 99% confidence are shown. Annotations about HtpG protein and peptides are from the NCBI database.

## Abbreviations

ACN, acetonitrile; BCG, *bacillus* Calmette-Geurin; Cas, CRISPR-associated protein; CRISPR, clustered regularly interspaced short palindromic repeat; CrRNA, CRISPR RNA; crRNP, crRNA ribonucleoprotein; eGFP, enhanced green fluorescent protein; EspB, Esx-1 secreted protein B; Esx-1, ESAT-6 secretion system-1; HtpG, high-temperature protein G; TLR-2, Toll-like receptor 2; IFN-γ, interferon gamma; IL, interleukin; LC, liquid chromatography; MS, mass spectrometry; MTB, *Mycobacterium tuberculosis*; PBS, phosphate-buffered saline; PBST, phosphate-buffered saline with tween 20; RNAP, RNA polymerase; SDS-PAGE, sodium dodecyl sulfate-polyacrylamide gel electrophoresis; SPR, surface plasmon resonance; TthCsm, Thermus thermophilus Csm.
