# Supplementary figures and images for: EspB and HtpG interact with the type III-A CRISPR/Cas system of Mycobacterium tuberculosis
Source: Front Mol Biosci. 2023 Nov 27;10:1261613. doi: 10.3389/fmolb.2023.1261613 (PMC10715591; doi:10.3389/fmolb.2023.1261613)

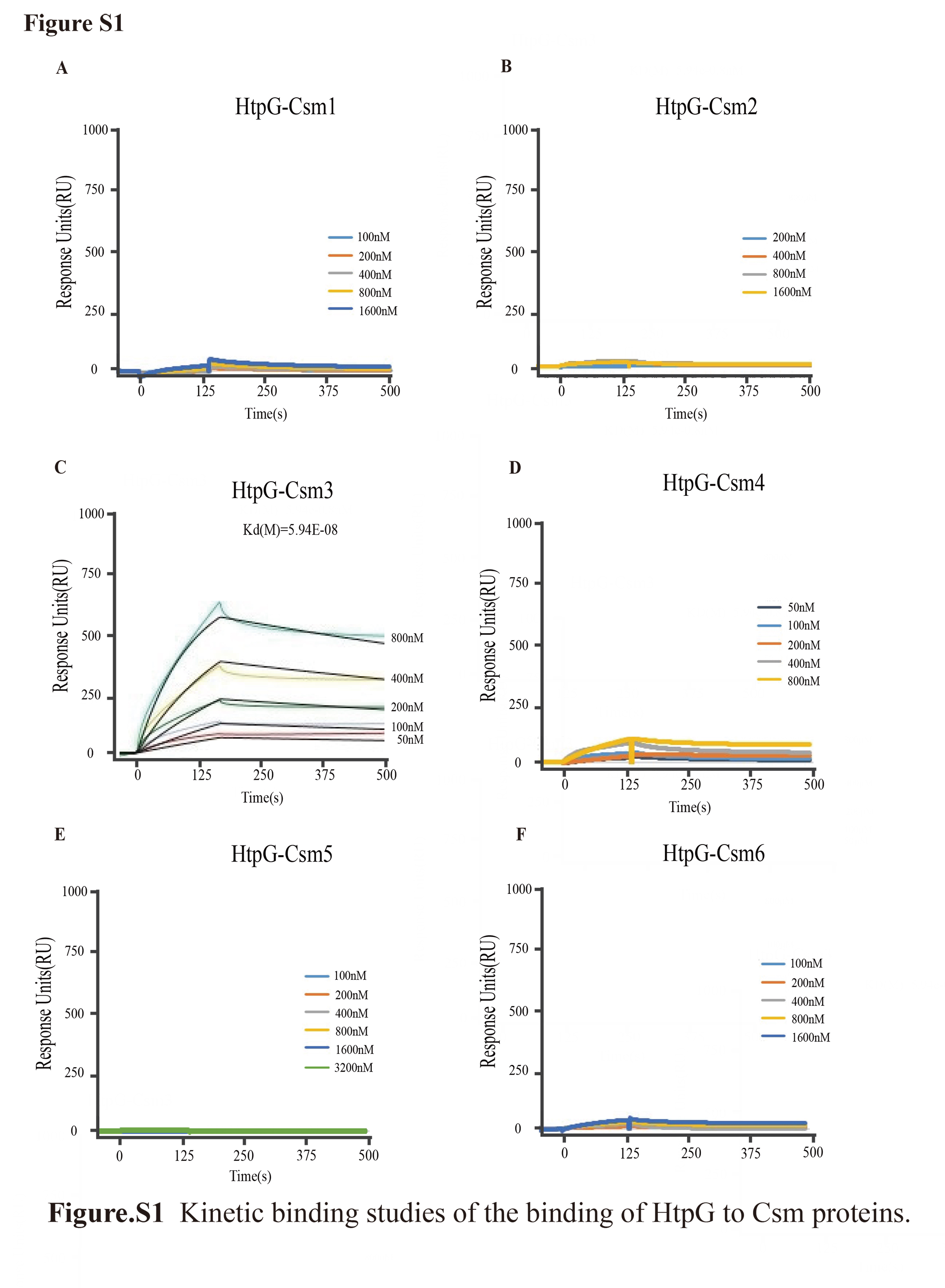

Supplement: Supplementary file 3 [file Image1.JPEG]

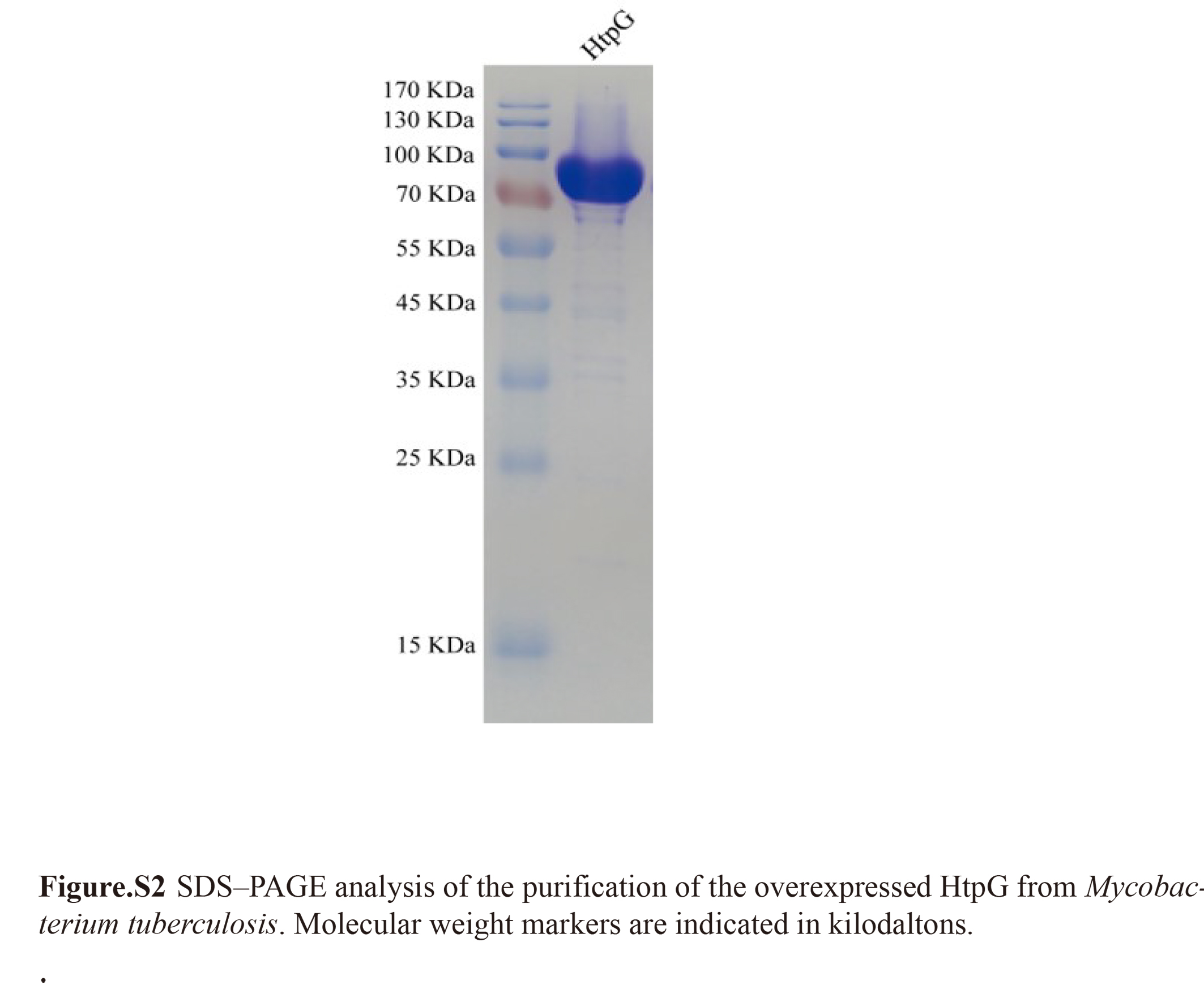

Supplement: Supplementary file 4 [file Image2.JPEG]
